# MicroRNA-99a and 100 mediated upregulation of FOXA1 in bladder cancer

**DOI:** 10.18632/oncotarget.2221

**Published:** 2014-07-15

**Authors:** Ross M. Drayton, Stefan Peter, Katie Myers, Saiful Miah, Ewa Dudziec, Helen E. Bryant, James W. F. Catto

**Affiliations:** ^1^ Academic Unit of Molecular Oncology, University of Sheffield, UK; ^2^ Academic Urology Unit, University of Sheffield, UK

**Keywords:** FOXA1, Urothelial cancer, Bladder cancer, FGFR3

## Abstract

Urothelial cell carcinoma of the bladder (UCC) is a common disease often characterized by FGFR3 dysregulation. Whilst upregulation of this oncogene occurs most frequently in low-grade non-invasive tumors, recent data reveal increased FGFR3 expression characterizes a common sub-type of invasive UCC sharing molecular similarities with breast cancer. These similarities include upregulation of the FOXA1 transcription factor and reduced expression of microRNAs-99a/100. We have previously identified direct regulation of FGFR3 by these two microRNAs and now search for further targets. Using a microarray meta-database we find potential FOXA1 regulation by microRNAs-99a/100. We confirm direct targeting of the FOXA1 3′UTR by microRNAs-99a/100 and also potential indirect regulation through microRNA-485-5p/SOX5/JUN-D/FOXL1 and microRNA-486/FOXO1a. In 292 benign and malignant urothelial samples, we find an inverse correlation between the expression of FOXA1 and microRNAs-99a/100 (r=−0.33 to −0.43, p<0.05). As for FGFR3 in UCC, tumors with high FOXA1 expression have lower rates of progression than those with low expression (Log rank p=0.009). Using global gene expression and CpG methylation profiling we find genotypic consequences of FOXA1 upregulation in UCC. Genetic changes are associated with regional hypomethylation, occur near FOXA1 binding sites, and mirror gene expression changes previously reported in FGFR3 mutant-UCC. These include gene silencing through aberrant hypermethylation (e.g. IGFBP3) and affect genes characterizing breast cancer sub-types (e.g. ERBB2). In conclusion, we have identified microRNAs-99a/100 mediate a direct relationship between FGFR3 and FOXA1 and potentially facilitate cross talk between these pathways in UCC.

## INTRODUCTION

Urothelial carcinoma of the bladder (UCC) is the fourth commonest male malignancy [1]. Despite its high prevalence and the cost of managing affected patients, there have been few advances in the treatment of this disease since the introduction of cisplatin based regimens [2, 3]. Clinical and molecular data suggest UCC is characterised by two distinct phenotypes with low and high-grade differentiation [4, 5]. Whilst the former has an excellent prognosis [6], high-grade tumors are aggressive cancers that progress to invasion, metastases and death [7]. Recent profiling reports have revealed invasive high-grade UCC can be clustered according to genes reflecting FGFR3 status (including papillary histology and FGFR3 related microRNAs), those seen in luminal breast cancer, and epithelial lineage and stem/progenitor cytokeratins (including a proportion of tumors with squamous histology) [8]. This clustering can reflect basal-like and luminal compartments in breast cancer, and may result in different sensitivities to cisplatin based chemotherapy [9], [10].

These data underlie the importance of FGFR3 in UCC biology. Whilst mutation of this oncogene occurs most commonly in low-grade non-invasive UCC [11], FGFR3 upregulation characterises the large chemosensitive sub-group of invasive cancers [10]. We were the first group to report FGFR3 upregulation in UCC through loss of microRNA-99a/100 expression (miRs-99a/100) [12]. We identified this appeared before FGFR3 mutation and may explain the high proportion of invasive UCC with upregulated wild type FGFR3 [8]. Importantly, the FGFR3 cluster of invasive UCC are also characterised by loss of expression of miRs-99a/100. MicroRNAs (miRs) are short non-coding RNA molecules that post-transcriptionally modulate protein expression [13]. Mature miRs are directed to mRNAs with a complementary seed sequence to nucleotides 1-8 of their 5' structure. Modulation of protein expression appears proportional to the extent of complementation, the context, the frequency and proximity, and the evolutionary conservation of the seed sequence. As each miR may target many mRNAs, we hypothesized that miRs-99a/100 modulate the expression of other mRNAs important for UCC biology. Here we report a search for and validation of potential candidates.

## RESULTS

### Identification of potential targets for miRs-99a/100

We extracted mRNA expression profiles from 7 microarray datasets [14-18] [19] [20] defining genes upregulated in n=958 low grade, superficial or non-muscle invasive UCC (n=12 Oncomine concepts, Supplementary table 1) when compared to either invasive (n=446) tumors or normal urothelial (n=135) samples. We selected the top 10% of upregulated genes (4,935 unique genes) and identified 20 that were predicted to be targets of miRs-99a/100 (from a total of 52 predicted targets). Upregulated genes appeared enriched for miR-99a/100 targets, when compared to the entire genome (20/4,935 vs 52/32,000, χ2=8.2, p<0.001). We ranked genes according to frequency of detection in the microarray comparisons (table 1). The commonest detected target was FGFR3 (found in 8/12 concepts), followed by the transcription factors FOXA1 and HOXA1 (in 6/12 and 3/12 concepts, respectively). We correlated expression profiles for miRs-99a/100 with these predicted targets using RNA profiles in the 72 samples from cohort 1 (table 2) and identified significant inverse correlations for FGFR3 (r=−0.48 to −0.52, p<0.01) and FOXA1 (r=−0.33 to −0.43, p<0.05). Non-significant negative correlations were seen for HOXA1.

**Table 1:** Predicted targets of microRNA-99a/100 found with increased expression in low grade or non-invasive in Urothelial carcinoma of the bladder The frequency of detection (from 12 Oncomine concepts) is shown, together with the Pearson's correlation coefficient to the expression of miRs-99a/100 in 72 urothelial samples

	Freq. in Oncomine concepts	Pearson's correlation of miR-mRNA expression	

Gene		miR-99a	miR-100
FGFR3	8	−0.48**	−0.52**
FOXA1	6	−0.43**	−0.33*
HOXA1	3	−0.22	−0.16
BMPR2	2	0.03	0.10
ICMT	2	0.07	0.05
MTMR3	2	0.04	0.05
OGT	2	0.04	0.05
ZBTB7A	2	−0.11	−0.05
ADCY1	1	−0.09	0.12
C4orf16	1	0.32	0.14
EIF2C2	1	−0.18	−0.12
FRAP1	1	−0.01	0.04
HS3ST3B1	1	0.08	0.08
IGF1R	1	−0.06	−0.07
NXF1	1	0.12	0.07
PI15	1	0.05	0.10
PPP1CB	1	0.04	0.13
SMARCA5	1	0.07	0.02
SMARCD1	1	0.18	0.04
ZZEF1	1	0.20	0.03

* Significance p<0.05

** Significance p<0.01

**Table 2:** Description of the patient samples used to investigate miRs-99a/100 targeting of mRNAs in bladder cancer The first cohort was used to investigate correlations between potential target genes and microRNA expression. The second cohort was used to explore the role of the predicted targets across the UCC spectrum

		Cohort 1		Cohort 2	
		n	%	n	%
Tissue	UCC	52	72.2%	207	94.1%
	Normal (UCC case)	10	13.9%	7	3.2%
	Normal (non-UCC)	10	13.9%	6	2.7%
Gender	Male	40	76.9%	161	73.2%
	Female	12	23.1%	59	26.8%
Age	Mean	72.3 yrs		71.9 yrs	
	Range	46-90 yrs		36-93 yrs	
Phenotype	Low Grade NMI	22	42.3%	60	27.3%
	High Grade NMI	12	23.1%	56	25.5%
	Invasive	18	34.6%	81	36.8%
	Not known			8	3.6%
Stage	pTa	25	48.1%	74	33.6%
	pTis	2	3.8%	9	4.1%
	pT1	7	13.5%	35	15.9%
	pT2-4	18	34.6%	81	36.8%
	Not known			8	3.6%
Recurrence	Yes	27	51.9%	49	22.3%
	No	25	48.1%	149	67.7%
Progression	Yes	18	34.6%	61	27.7%
	No	34	65.4%	137	62.3%
Follow up	Mean	35.6 months		32.4 months	
	Range	0-93 months		0.6-111 months	
Total		72	100%	220	100%

### Targeting of FOXA1 by miRs-99a/100

**Figure 1:**
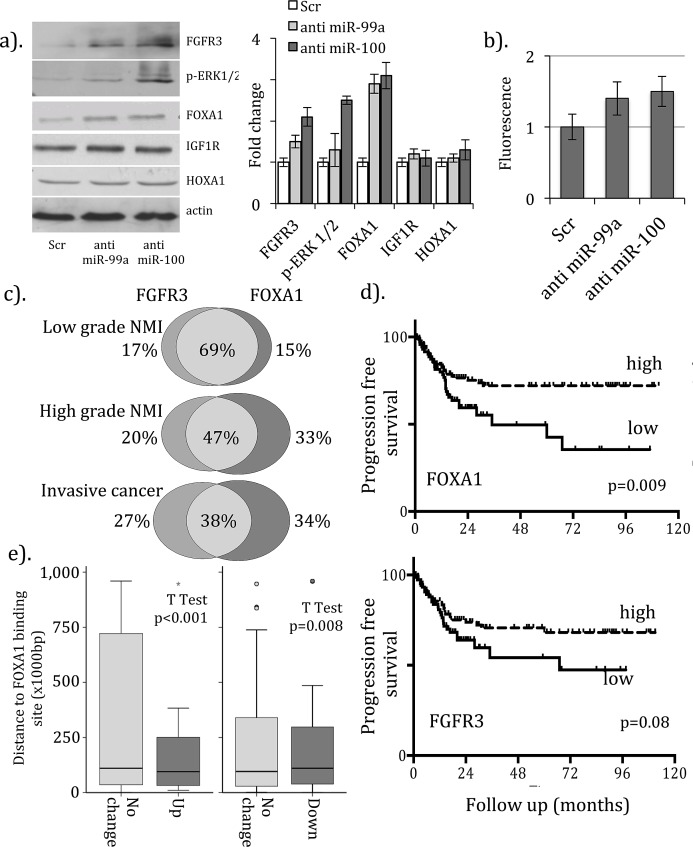
Regulation of FOXA1 expression by microRNAs-99a/100 in bladder cancer (a). Western blotting for protein expression reveals upregulation of FGFR3, phosphorylation of ERK1/2 and FOXA1 following transfection of NHU cells with anti-miRs to miRs-99a/100 but not the scrambled RNA control. Image densitometry reveals 3-fold increases in expression for FOXA1 with anti-miRs-99a/100. (b). A reporter construct assay reveals 1.3/1.4 fold increases in luciferase expression in NHU cells treated with anti-miRs-99a/100, when normalized to the scrambled control. Expression profiling using QrtPCR in 220 benign and malignant urothelial samples revealed (c). overlap of upregulation for FOXA1 and FGFR3 in UCC, which is greatest in low-grade NMI cancers, and (d). lower rates of progression to more advanced disease for tumors with high FGFR3 and high FOXA1 when compared to those with low expression (the difference in greatest in low stage and low tumors (supplementary figure 3)). (e). Genes with upregulation following FOXA1 transfection in T24/EJ cells are located closer to known FOXA1 binding sites (in HepG2 cells) than those with no change in expression or those with reduced expression (down regulation).

To explore targeting of FOXA1 and HOXA1 by miRs-99a/100, we examined protein expression in NHU cells following microRNA knockdown. For comparison we included FGFR3, phosphorylated-ERK 1/2 (as a marker of FGFR3 signaling pathway activity) and IGF1R (also potential target). Upregulation of FOXA1 (2.9 to 3.1 fold (±st. dev. 0.23 to 0.32)) and FGFR3 (1.5 to 2.1 fold (±0.15 to 0.23)) and ERK1/2 phosphorylation (1.3 to 2.5 fold (±0.1 to 0.4)) was seen following knockdown (figure 1a). In contrast, no change was seen for IGF1R and HOXA1. To investigate direct targeting of the FOXA1 3' UTR we synthesized a luciferase reporter construct incorporating the miR-99a/100 seed sequence from FOXA1. We observed increases in luciferase fluorescence with anti-miRs to miR-99a (1.4 fold (±0.2)) and miR-100 (1.5 fold (±0.21), figure 1b).

Whilst direct targeting of the FOXA1 3' UTR may occur through miRs-99a/100, we wondered whether a further indirect pathway may contribute to the 2-3 fold rise in protein expression seen. We investigated the potential for microRNA networks within a tumor [21]. We performed TLDA multiplex qrtPCR in triplicate on the NHU cells with anti-miRs to 99a/100 and a scrambled control. When normalized to this control, concordant reduced expression (<0.5 fold) was seen for 9 miRs (7 shared between miRs-99a/100) and increased expression (>2 fold) for 63 miRs (supplementary figure 1). We focused upon the 7 miRs with reduced expression for both miRs-99a/100, hypothesizing their loss could lead to upregulation of FOXA1 promoting factors. The expression of each miR was closely correlated (Pearson's Coefficient r=0.43 to 0.74, p=8.0×10^−14^ to 0.08, table 3) with miRs-99a/100 in the 72 urothelial samples from cohort 1. Predicted mRNA targets of these 7 miRs (obtained from TargetScan) included 14 transcription factors with binding sites within the FOXA1 promoter, e.g. miR-485-5p predicted to target SOX5, JUN-D and FOXL1, and miR-486 proven to regulate FOXO1a [22]. As such, loss of expression of these miRs may lead to upregulation of these transcription factors and increased FOXA1.

**Table 3:** Correlation of microRNAs with loss of expression following miRs-99a/100 knock-down (a). Expression is shown (as fold change) for each miR in NHU cells transfected with anti-miRs to miRs-99a/100, normalised to scrambled RNA control. For each, the correlation of expression is also shown in 72 urothelial samples (from cohort 1). In (b). we show predicted targeting by these microRNAs of transcription factors with binding sites within the FOXA1 promoter. For example, miR-485-5p is predicted to target 3 transcription factors. Targeting of FOXO1a by miR-486 has been reported [22]

a). Reciprocal loss of microRNA expression following miRs-99a/100 knock-down							
	miR-485-5p	miR-500a	miR-486	let-7e	miR-657	miR-133b	miR-139-5p
Fold change (with anti miR-100)	0.001	0.01	0.01	0.04	0.04	0.001	0.05
Fold change (with anti miR-99a)	0.001	0.01	0.06	0.27	0.33	0.99	0.05
Correlation with miR-100 (Pearson, R)	0.71	0.52	0.43	0.56	0.26	0.65	0.68
p value	2.01E-09	6.20E-06	4.98E-05	5.14E-08	9.52E-02	4.03E-11	2.38E-11
Correlation with miR-99a (Pearson, R)	0.68	0.57	0.46	0.6	0.27	0.65	0.74
p value	1.61E-08	7.60E-07	1.23E-05	4.61E-09	8.87E-02	4.12E-11	8.00E-14
b). Transcription factors within the FOXA1 promoter							
	miR-485-5p	miR-500a	miR-486	let-7e	miR-657	miR-133b	miR-139-5p
AHR		1					
AML1A		1					
BACH1				1			
FOXL1	1						
FOXO1a			1				1
HOXA9				1		1	1
JunD	1						
SOX5	1						1
Sp1					1	1	

### FOXA1 and FGFR3 expression in bladder cancer

Whilst recent data report upregulation of both FGFR3 and FOXA1 characterize the papillary (luminal breast cancer-like) molecular subtype of invasive UCC [8] [10], these reports did not include low-grade tumors. To examine expression throughout the UCC spectrum and to compare profiles with outcome, we measured mRNA using QrtPCR in 220 urothelial samples (cohort 2). For both mRNAs, highest expression was seen in non-invasive pTa and low-grade tumors (ANOVA p<0.02, Supplementary figure 2)). Upregulation of both FGFR3 and FOXA1 (when dichotomized around the mean) was seen in 69% of low-grade, in 47% of high-grade NMI and in 38% of muscle invasive cancers with high expression of either (figure 1c, χ2=18.0, p<0.001). Tumors with high expression had lower rates of progression to more advanced disease, when compared to those with low expression (figure 1d). This difference reached significance for FOXA1 (Log rank p=0.009, Bonferroni corrected) but not FGFR3 (p=0.08). Sub-group analysis revealed that this difference in progression free survival was only apparent in non-invasive cancers (supplementary figure 3) and in low grade tumors (data not shown, Log rank values for FOXA1; low grade p=0.002, high grade 3 p=0.24. Log rank values for FGFR3; low grade 1 p=0.003, high grade 3 p=0.29).

### Molecular events of FOXA1 upregulation in Bladder Cancer

Having identified high FOXA1 and high FGFR3 expression are correlated in a cohort of UCC and partially regulated through miRs-99a/100, we wondered about the molecular consequences of FOXA1 upregulation in UCC. For analysis, we transfected EJ/T24 cells (as they have low FOXA1 expression, have wild type FGFR3 and are not dependent upon FGF signaling) with FOXA1 or the empty plasmid (control)(supplementary figure 4) [23]. We analyzed whole genome mRNA expression and CpG methylation, as reports show that FOXA1 recruitment is associated with DNA demethylation and changes in chromatin conformation [24-26] [27]. Microarray data were filtered for probes concordant between duplicates and experimental replicates, before matching between platforms and normalizing to control (cells transfected with the empty plasmid). Our final dataset included 12,939 genes with 162,338 matching CpG probes across the promoter region (n=51,112), inside exon 1 and around transcription start sites (n=105,133), and downstream from the gene (n=6,093) (dataset is available at http://www.sheffield.ac.uk/oncology/units/urology/data). As expected, we observed increases in gene expression with the density of CpG hypomethylation and reductions in expression with hypermethylation (T test p<0.001, supplementary figure 5) in FOXA1 transfected cells [28]. These associations were closest for CpG probes within the gene promoter and around the transcription start site/exon 1 (annotated as inside the gene), when compared to probes downstream from the gene.

FOXA1 transfection produced upregulation of 5,455/12,939 (42%) transcripts including 1,650 (13%) with more than 1.2 fold increase (supplementary figure 5), when compared to controls. Differences in expression reached significance (FDR <5%) in 50 genes, including 14 up and 36 down-regulated mRNAs. These were involved in the negative regulation of signal transduction, chromatin alterations and nucleosome assembly, DNA methylation, DNA binding and mutagenesis repair and metabolic regulation (p<0.05, supplementary table 2). Identified members included those previously shown to be aberrantly silenced through DNA hypermethyaltion in UCC, e.g. IGFBP3 [29], and those found to charactize breast cancer sub-types (e.g. ERBB2) [10]. MEDIP-Chip identified increases in CpG hypomethylation for 32/111(29%) promoter, 32/96 (33%) inside and 10/13 (77%) downstream probes (chi sq. p=0.002) within 5 of the 14 upregulated genes. Contrastingly, reduced mRNA expression was seen in 7,484 (58%) genes following FOXA1 transfection, including 567 (4%) with less than 0.8 fold change. Increases in aberrant hypermethylation were seen at CpG loci in 1,708 (23%) and 134 (24%) of these down regulated genes, respectively. Motallebipour et al. mapped FOXA1 binding sites in the hepatocellular carcinoma cell line HepG2 [30]. We annotated our dataset with these loci and compared proximity for these 50 significantly altered genes. Genes with increased expression following FOXA1 transfection were significantly closer to FOXA1 binding sites (mean 217,515bp (95%CI=159,432-275,598)) than those without change (1,774,996bp (1,110,313-2,439,680), figure 1e, T Test p<0.001). Conversely, those with reduced expression following FOXA1 transfection were significantly more distant (2,048,425bp (1,015842-3,081,008) than those without change (802,277bp (425,626-1,178,928) p=0.008).

### FOXA1 gene expression profiles in FGFR3 mutant and sporadic bladder tumors

**Figure 2:**
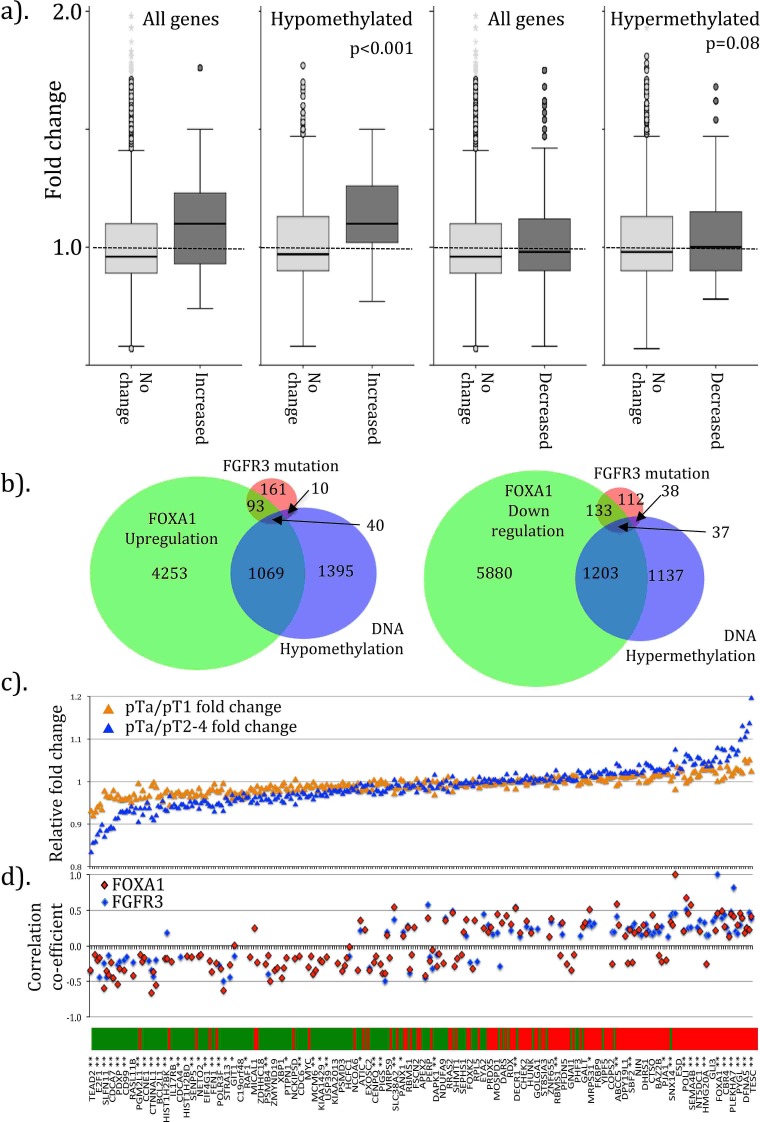
Symmetry of genotype between FOXA1 transfected cells, FGFR3 mutant tumors and in sporadic UCC Gene expression changes following FOXA1 transfection in T24/EJ cells share many similarities with those found in FGFR3 mutant bladder UCC. In (a). we stratify changes in normalized gene expression following FOXA1 transfection within T24/EJ by those seen in FGFR3 mutant UCC. Genes upregulated in FGFR3 mutant tumors (labeled as Increased) have significantly higher expression in FOXA1 transfected cells than those with no change in FGFR3 mutants. The difference is largest for genes with associated DNA hypomethylation. No significant difference is seen for genes with decreased expression or DNA hypermethylation. (b). Area proportional Venn diagrams reveal the overlap for genes in FGFR3 mutant UCC and those upregulated following FOXA1 transfection or with increases in DNA hypomethylation following FOXA1 transfection (left). Overlap was also seen for genes downregulated in FGFR3 mutant UCC, and those down regulated or hypermethylated following FOXA1 transfection. We identified a 156 gene cohort with reciprocal, symmetrical expression in FOXA1 transfected cells and FGFR3 mutant UCC and analyzed this in a publically deposited microarray dataset [32]. In (c). we plot the fold change of these 156 genes as a ratio for low-grade NMI cancers relative to high-grade NMI cancer (orange triangle) and invasive cancers (blue). In (d). we plot the correlation coefficient vales (r) between expression of FGFR3 and FOXA1 in the 256 sporadic UCC [32]. The 156 genes in (c) and (d) are ordered according to expected fold changes (bar: green is loss of expression, red is increased expression) as seen in FOXA1 transfected cells. Significance of difference in fold change (low grade NMI versus more aggressive cancers) is shown as *p<0.05 and **p<0.01.

Our data report correlated upregulation of FOXA1 and FGFR3 in UCC, partly through dual regulation by miRs-99a/100. Consequently, tumors with aberrant FGFR3 or FOXA1 expression may have symmetry of genotype. Lindgren et al. reported 380 genes upregulated and 468 down regulated in 46 UCC with mutant FGFR3 compared to 29 wild type controls [31]. We identified 222 and 325 of these, respectively, in our dataset. When compared, genes with upregulation in FGFR3 mutant cancers had significantly higher expression in the FOXA1 transfected cells (average 1.10±0.2 fold change, p<0.001, Figure 2a) than those without change. The difference was greater when genes with probes enriched for CpG hypomethylation were selected (relative fold change 1.12±0.2, p<0.001). In contrast, no difference in expression was seen for genes down regulated in FGFR3 mutant cancers in our FOXA1 transfected cells, even if those with hypomethylated or hypermethylated probes were selected (fold change 1.03±0.17, p=0.08). Comparison between the genes altered with FGFR3 mutation, with FOXA1 transfection and changes in DNA methylation revealed considerable overlap (figure 2c). Genes with increased expression in FGFR3 mutant cancers were significantly closer to FOXA1 binding sites (433,101bp (95%CI=331,878-534,324)) than those without change (837,209bp (814,843-859,575) T Test p<0.001). Those with reduced expression in FGFR3 mutants were also significantly closer (315,103bp (261,634-368,572)) than those without change (841,086bp (818,606-863,570), p<0.001).

For external validation, we examined the genes with symmetrical changes in FGFR3 mutant UCC and FOXA1 transfected cells (135 up and 170 down regulated) using a large microarray dataset of 256 sporadic UCC [32]. We identified 295 (97%) members and found significant aberrant expression, in the symmetrical manner seen in FOXA1 transfected cells and FGFR3 mutant UCC, for most gene members (89 (67%) up and 87 (54%) down-regulated genes, T Test p<0.05, suppl. figure 8a). There was significant correlation of expression between FOXA1 and FGFR3 (r=0.45 (95%CI 0.35-0.55), p<0.001), and once again the expression of each was highest in non-muscle invasive cancers (suppl. figure 8b, Χ^2^ p<0.01). Comparative analysis revealed 156/295 (54%) of these genes were significantly differentially expressed between low-grade NMI, high-grade NMI or invasive cancers (Figure 2c, t test p<0.05) in the manner expected from FGFR3 mutant UCC/FOXA1 transfected cells. The expression of many genes was significantly correlated with FGFR3, FOXA1 or both. In particular, 72 and 62 predicted up regulated genes were significantly positively correlated to increases in FOXA1 (r=0.12 to 0.82) and FGFR3 expression (r=0.12 to 0.82, Figure 2d). Conversely, 32 and 77 predicted down regulated genes were significantly negatively correlated with FOXA1 (r=−0.14 to −0.51, p<0.05) and FGFR3 (r=−0.12 to −0.66, p<0.05) expression, respectively.

## DISCUSSION

Here we report FOXA1 as a regulatory target of miRs-99a/100 in UCC. This follows our previous findings identifying FGFR3 as a target of miRs-99a/100 [12] and supports data revealing these microRNAs discriminate of UCC genotype [8]. Our work provides a direct mechanistic link between miRs-99a/100, FGFR3 and FOXA1. The central role of miRs-99a/100 in this association suggests epigenetic events either precede the development of tumor genotype or act as a link between these pathways in tumors when either event occurs in isolation. For example, FGFR3 mutation may lead to increased mRNA expression, annealing and sequestration of miRs-99a/100 leading to FOXA1 upregulation [33]. There was considerable overlap between the genotypes of tumors with FOXA1 upregulation and FGFR3 mutation. This symmetry appears mediated through miRs-99a/100 and FOXA1, given the proximity of affected genes to FOXA1 binding sites and to changes in DNA hypomethylation, suggesting this axis plays a key role in determining genetic events in low-grade and papillary-type invasive high-grade UCC. Genotype often impacts upon phenotype, and we observed tumors with high expression of both FOXA1 and FGFR3 were most commonly low grade non-invasive and had lower rates of progression, when compared to those with low expression. Outcome differences were only seen in non-invasive cancers, suggesting consequential changes subsequent to these genes become more important with tumor evolution.

Our data provide an explanation of previous CpG methyl profiling reports. Wolff *et al.* identified regional hypomethylation across 16% of CpG probes in low grade UCC, when profiling a large UCC cohort [34]. FOXA1 is a transcription factor recruited to enhancers that acts to influence chromatin interactions through DNA demethylation and H3K4 methylation [24, 27]. The role of FOXA1 in malignancy has been best studied in endocrine-dependent cancers, where it is known to act as a transcriptional co-factor and to modulate hormone receptor activity through chromatin and DNA modifications [35].

Our findings also reflect those in a previous report of FOXA1 in UCC [36], although we make a different interpretation. Specifically, we propose microRNA-mediated upregulation of FOXA1 in papillary type UCC, rather than loss of FOXA1 expression in aggressive, squamous tumors. Our findings mirror those seen in other malignancies, e.g. FOXA1 upregulation confering a good prognosis on cancers with high expression [37, 38] and is a representative marker of luminal-type cancers [39], and are compatible with DeGraff et al. given recent evidence of a dual role for up and down regulation of FOXA1 in cancer [40, 41].

In summary, we identify microRNA mediated upregulation of both FGFR3 and FOXA1 in UCC. We propose this is a determinant of the papillary genotype of these cancers and that FOXA1 is a key mediator of this evolution.

## MATERIALS AND METHODS

### In Silico identification of miR-99a/100 targets in UCC

To identify potential targets of miRs-99a/100 in low grade UCC we created a microarray meta-database from publically deposited UCC mRNA expression datasets (from www.oncomine.org). We selected datasets in which we could identify genes upregulated in low grade, superficial or non-muscle invasive UCC, when compared to controls and/or high grade or invasive tumors. We searched for predicted targets of miRs-99a/100 (obtained from TargetScan (Version 4.2, www.targetscan.org) and PicTar (http://pictar.mdc-berlin.de)) across this meta-database and ranked according to the frequency of detection.

### Patients and tumours

To evaluate potential mRNA targets we studied 292 freshly frozen urothelial samples (Table 1) from two cohorts. The first was used to compare the expression of miRs-99a/100 and putative mRNA targets. The second examined these putative targets in a larger unrelated population. UCC were classified using the 2004 WHO/ISUP criteria and treated according to standard care [5, 42]. Histologically normal urothelial samples were obtained from patients with UCC (distant to any tumor) and disease-free controls (at prostatectomy). We analyzed UCC cell lines representing the disease spectrum (RT4, RT112 and EJ/T24, respectively, purchased from ATCC) and normal non-immortalized human urothelial (NHU) cells [43].

### RNA extraction, cDNA synthesis and rtPCR

We extracted total RNA using the mirVana^™^ kit (Ambion, TX) from 10 x 10uM microdissected frozen tumor sections (>90% pure cell populations). We measured the expression of potential mRNA target using quantitative rtPCR (Taqman commercial assays purchased from Applied Biosystems, UK). cDNA was made using 100ug whole RNA, random primers, RT buffer, dNTP (100mM), RNase inhibitor and MultiScribe Reverse Transcriptase (cDNA Reverse Transcription kit, Applied Biosystems, Warrington, UK). Realtime quantified PCR with 2 μL cDNA, gene specific primers with FAM-TAMRA labeled probes, water and 2x Taqman Universal PCR MasterMix (Applied Biosystems, Warrington, UK) was performed on the ABI 7900HT system according to manufacturers guidelines. Relative mRNA quantification was determined with respect to the mean of GAPDH and β-Actin. We also used quantitative rtPCR to determine microRNA concentrations, with reagents specific to the mature 23bp sequence, in isolation and using the commercial Taqman low density microarray (n=365 miRs, as detailed in [12]). Reverse transcription using stem loop primers was performed with 50ng small RNA, MultiScribe Reverse Transcriptase (Applied Biosystems, Warrington, UK), RNase inhibitor, 100nm dNTPs and nuclease free water. Quantification with MGB labeled probes specific to the reverse transcribed product was performed according to the manufacturer's guidelines (Applied Biosystems, Warrington, UK). Relative miR quantification was determined with respect to the average of two snoRNAs (RNU44 and RNU48) or the mean of the entire TLDA.

### MicroRNA manipulation and Luciferase reporter construct

To examine functional implications of miR expression, we manipulated expression using specific anti-miRs and a scrambled RNA sequence controls (Ambion, TX). All experiments were performed in triplicate using non-immortalized NHU cells at 70% confluence, as detailed [44]. Briefly, each well of a six-well tissue culture plate was transfected with 100 pmol anti-miR using 5 μl siPORT (Life Technologies) in 200 μl Optimem (Life Technologies). Knockdown of relevant miR was confirmed by miR-specific taqman PCR (Applied Biosystems) after 48 hours.

To investigate direct targeting of the FOXA1 3'UTR we synthesized a Luciferase reporter construct (methods detailed [44]). We cloned 800 bases around the miR-99a/100 seed sequence in the FOXA1 3' UTR (chr14: 37,129,203 - 37,129,210) into EJ cells and ligated into pMIR REPORT (Invitrogen, UK). Dual luciferase assays were conducted in a 6 well plate format at 70% confluence. Forty-eight hours post transfection, firefly and renilla luciferase were quantified sequentially using the Dual Luciferase Assay kit (Promega, UK) and luminescence was measured using the manufacturers recommended luminometer (Promega Glomax). Firefly luciferase expression was quantified and normalized to Renilla luciferase expression.

### FOXA1 transfection

EJ cells were transfected with a pcDNA3 vector containing FOXA1 or an empty vector control. 24 μg DNA / 90 μl Lipofectamine LTX (Life Technologies) was used to transfect each 90mm dish at 70% confluence. Experiments were performed in triplicate. A small portion (20%) of transfected cells was used to confirm FOXA1 expression by western blot.

### Genome wide profiling of DNA methylation

Methylated DNA immunoprecipitation and tiling CpG island microarrays (Human CpG Island Microarray, Agilent, CA) (MeDIP-CHIP) were used to determine genome wide methyl-cytosine profiles, as detailed elsewhere [28]. Genomic DNA was sonicated and incubated with antibodies raised to either 5mC (anti-5-methylcytidine, Eurogentec, Hampshire, UK) or murine IgG (negative control). The antibody-antigen complex was captured with magnetic beads conjugated to anti-mouse-IgG (Santa Cruz Biotechnology), washed, unbound, non-specific DNA removed, before methylated DNA elution. Immunoprecipitated (Cyanine 5-dUTP) and reference DNA (Cyanine 3-dUTP) were labeled (Genomic DNA Enzymatic Labeling Kit, Agilent), cleaned (Amicon filters, Millipore) and quantified. Competitive hybridization onto the CpG microarray was performed (ChIP-on-Chip Hybridization Kit, Agilent) in a rotating SureHyb chamber at 67°C for 40 hours. Washed slides were scanned (High-Resolution C Scanner, Agilent) and fluorescence obtained using Feature Extraction software. The microarray contains 244,000 probes that tile through 27,800 CpG features at an average of 100bp separation. We identified concordant probes within the highest (>80%) and lowest quintiles (<20% of Cy-5) fluorescence and defined these as enriched for hyper or hypo-methylated, respectively. Finally, as each experiment was performed in triplicate, we excluded probes without concordance in 2 or 3 replicates.

### Whole genome mRNA expression

Whole genome mRNA expression was determined by microarray (HG-U133 Plus 2.0, Affymetrix, Cal.) [28]. This platform contains 54,000 probesets, including 33,000 to known coding genes. RNA was prepared using the Affymetrix protocol (enzymes from Invitrogen) and annealed to an oligo-d(T) primer with a T7 polymerase binding site. cDNA was generated using superscript II and E. coli DNA ligase and polymerase I. The reaction was completed with T4 DNA polymerase and EDTA. Amplified cDNA was cleaned, biotin-labeled, fragmented and hybridized to the microarray for 16 hours at 45°C in a rotating oven at 60rpm. After washing and staining, the arrays were scanned (GC3000 scanner) and data processed using Gene Chip Operating System software. mRNA expression was determined using Microarray Analysis Suite 5 (Affymetrix) and defined as expressed (perfect match probeset intensity greater than mismatch intensity) or absent (mismatch probeset intensity greater or equal to perfect match intensity). Expression data were exported into Expression console (Affymetrix), RMA log2 converted and analyzed within Significance Analysis of Microarrays [45]. This microarray data is deposited on line at GEO datasets (GEO accession number GSE56037).

### Protein expression: western blotting

Cells were lysed in RIPA buffer (20m M Tris·HCl, 135 mM NaCl, 10% glycerol, 1% Igepal, 0.1% SDS, 0.5% deoxycholic acid, 2 mM EDTA) containing protease and phosphatase inhibitors (Complete EDTA-free protease inhibitor cocktail and PhosSTOP phosphatase inhibitor cocktail; Roche, Mannheim, Germany), and protein content was quantified using the DC-protein assay reagent (Bio-Rad, Hercules, CA). Protein lysates (50μg) were loaded onto 8% gels, fractionated, and electroblotted onto nitrocellulose membranes. After blocking with 5% non-fat milk powder and 0.1% Tween, the membranes were incubated overnight with the relevant primary antibody (listed in supplementary table 3) overnight at 4°C, washed, and incubated at room temperature for 1h with an appropriate HRP-conjugated secondary antibody (1:1000; Cell Signaling Technologies Inc.). The immune complexes were visualized by enhanced chemiluminescence (GE Healthcare, Buckinghamshire, UK) and quantified using Image J for band densitometry. The final values were plotted relative to the negative control and normalized to the corresponding Beta-Actin value.

### Statistical analysis

Relative mRNA and miR concentrations were calculated using the median of their respective reference molecules (ΔCt = Ct _miR_ – Ct _median control_) and expression fold changes computed using 2^−ΔΔCt^ calculations [46]. MiR-mRNA expression was correlated using Pearson's coefficient. RNA expression was compared with clinicopathological data using the χ2, T test or Mann Whitney U test where appropriate. Disease progression was defined when a non-muscle invasive tumor became invasive or a muscle invasive tumor developed metastases. Progression-specific survival probability following tumor resection was analyzed using the Kaplan-Meier method and compared with the Log rank test, for which significance was adjusted using a Bonferroni correction. Patients without progression were censored at last reviewed or when they died of other causes. All analyses were two tailed and carried out using SPSS (version 14, SPSS Inc).

## SUPPLEMENTARY MATERIAL FIGURES NAD TABLES




