# Supplementary material for: MicroRNA-99a and 100 mediated upregulation of FOXA1 in bladder cancer
Source: Oncotarget. 2014 Jul 15;5(15):6375–86. doi: 10.18632/oncotarget.2221 (PMC4171637; doi:10.18632/oncotarget.2221)
Supplement: Supplementary file 1 [file oncotarget-05-6375-s001.pdf]

MicroRNA-99a and 100 mediated upregulation of FOXA1 in bladder cancer

Supplementary Material

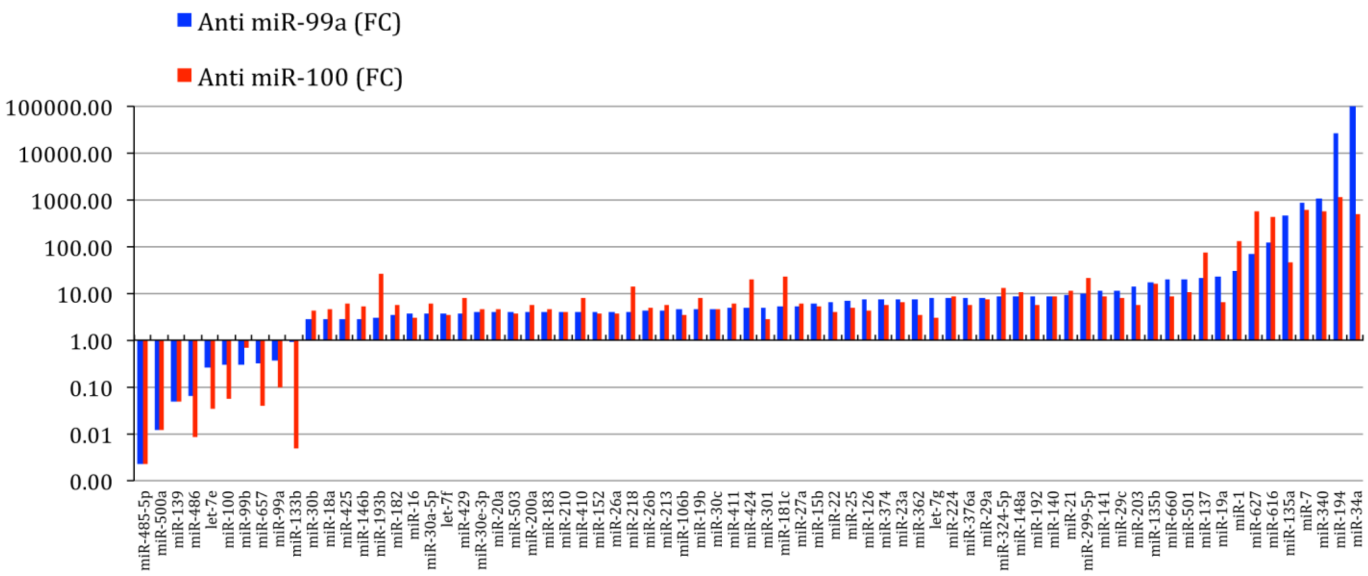

Supplementary figure 1: MicroRNA expression following knock-down of miR-99a and miR-100. Whilst most RNAs increase expression (those selected have >2 fold), a minority have reduced expression (defined as <0.5 fold).

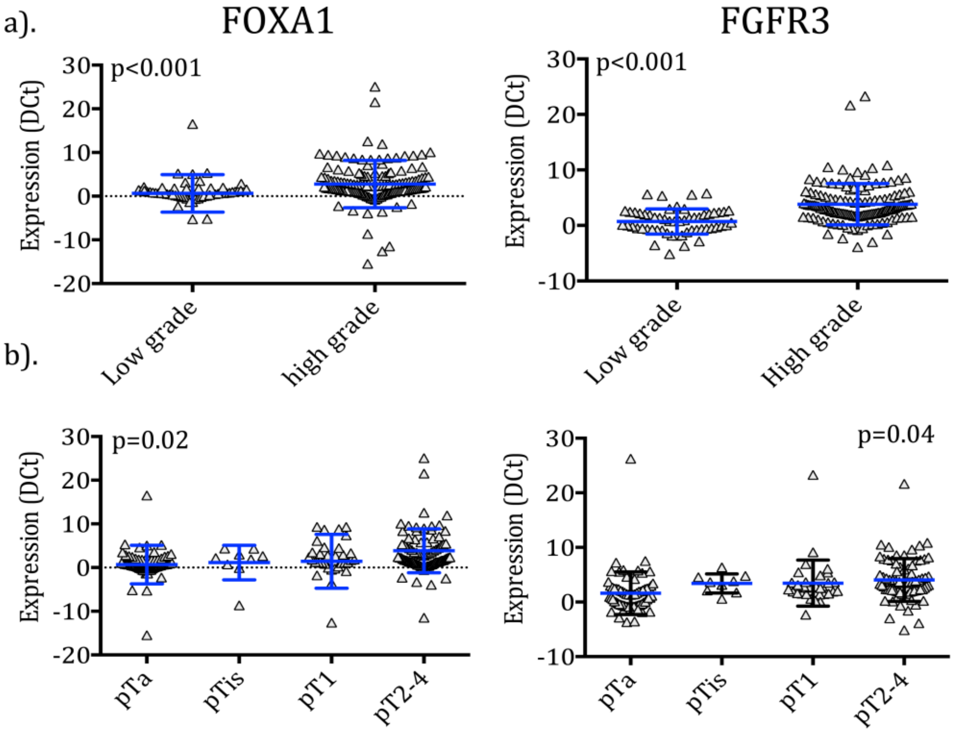

Supplementary figure 2: Expression of FOXA1 and FGFR3 mRNA in bladder cancer. Plots reveals the expression of each mRNA (shown as DCT values normalized to B-actin and GAPDH). For each, the highest expression was seen tumors of low stage and low grade.

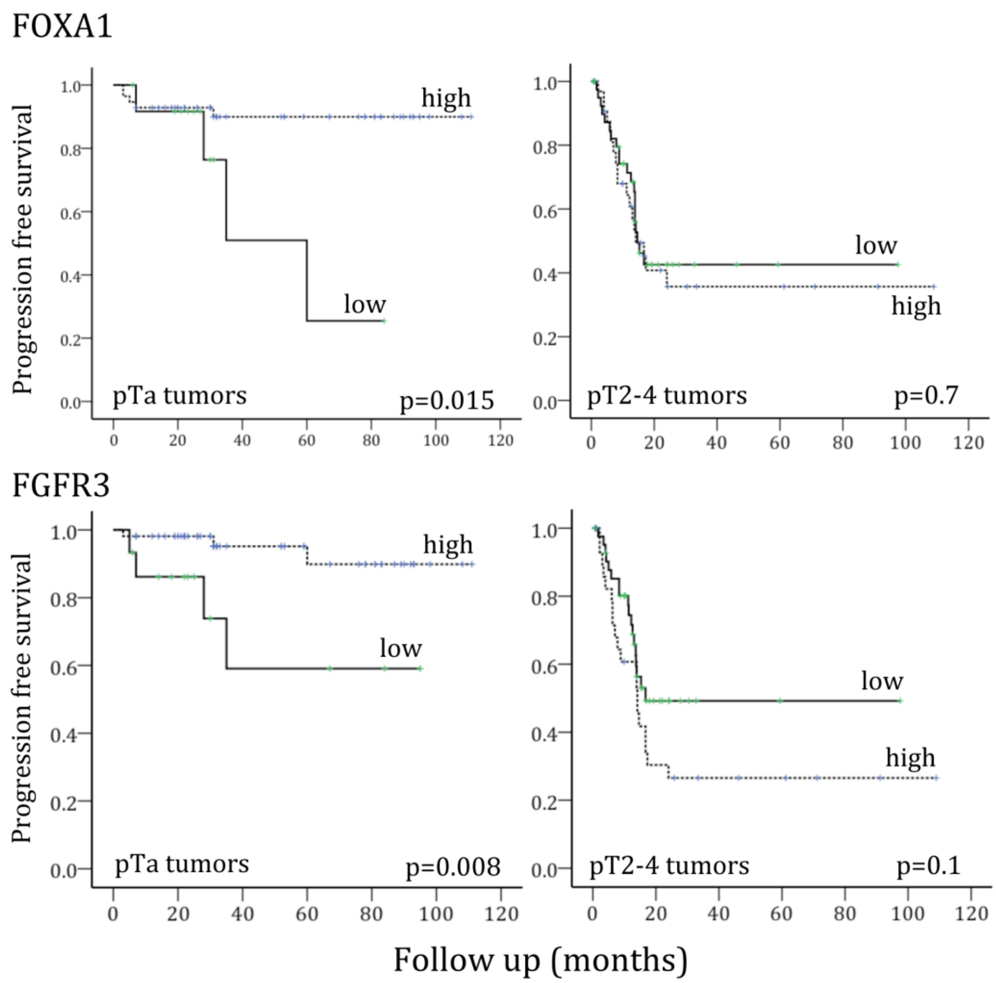

Supplementary figure 3: Progression free survival with respect to FOXA1 and FGFR3 expression for non-invasive (pTa) and muscle invasive (pT2-4) bladder cancers. Differences in progression to more advanced stage tumors were seen only in non-invasive tumors for both FOXA1 and FGFR3.

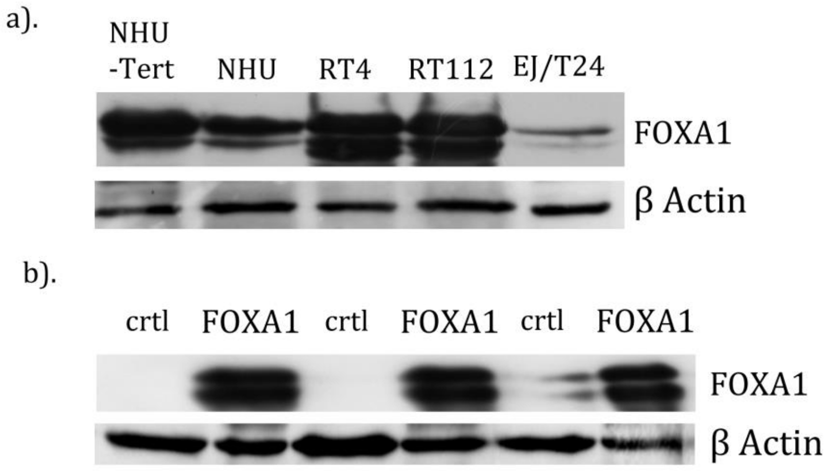

Supplementary figure 4: FOXA1 expression in bladder cancer cell lines. (a). Protein expression of FOXA1 is detected through Western blotting in normal urothelial cells (both immortalized; NHU-Tert, and Non-immortalized; NHU) and those representing the bladder cancer spectrum (RT4; low grade non-invasive (has amplification of non-mutant FGFR3 (Williams et al.))), RT112 (high-grade invasive (has amplification of non-mutant FGFR3)) and EJ/T24 (invasive cancer (wild type FGFR3)). Of note, RT4 and RT112 are sensitive to FGF receptor inhibition (Lamont et al.). (b). Transfection of FOXA1 into EJ/T24 cells (triplicates of negative controls (crtl) and FOXA1 transcripts.

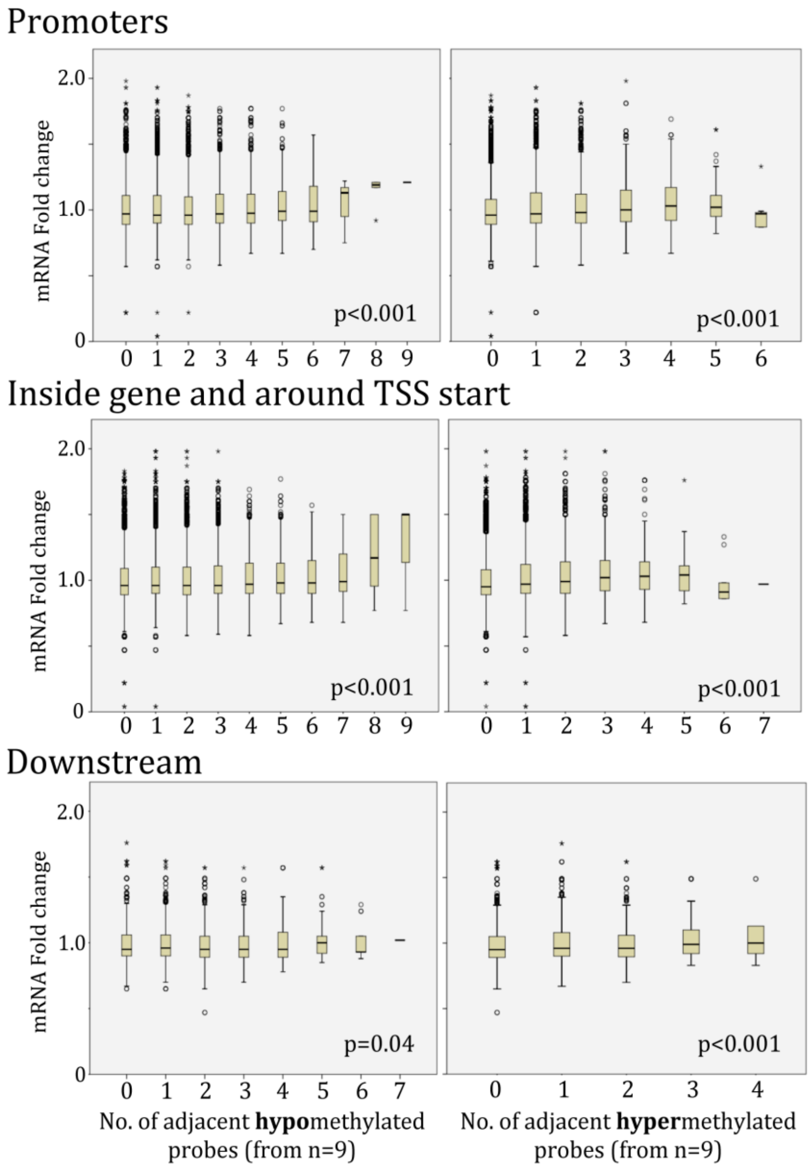

Supplementary figure 5: Changes in DNA methylation and gene expression with FOXA1 transfection. mRNA expression is plotted as relative fold change in FOXA1 transfected cells when compared to control cells for 13,067 genes, according to the number of adjacent CpG probes enriched for either hypo (left hand plots) or hyper (right hand plots). mRNA expression (boxes represent mean and 95% confidence intervals) increases with the number of adjacent hypomethylated probes and decreases with the number of hypermethylated probes most dramatically around gene promoters and inside the coding region (mostly within exon 1 and around ATG start site).

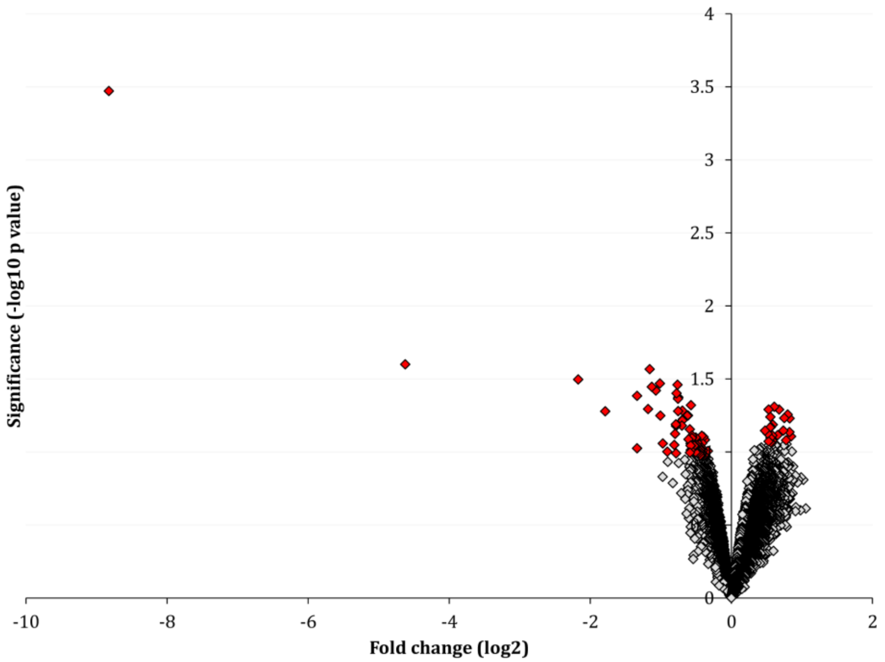

Supplementary figure 6: Changes in gene expression in bladder cancer cells with FOXA1 transfection. The volcano plot reveals global changes in gene expression determined from the whole genome mRNA expression microarrays in cells transfected with FOXA1, when compared to control cells with the empty plasmid. Those in red reached significance in SAM (FDR <5%).

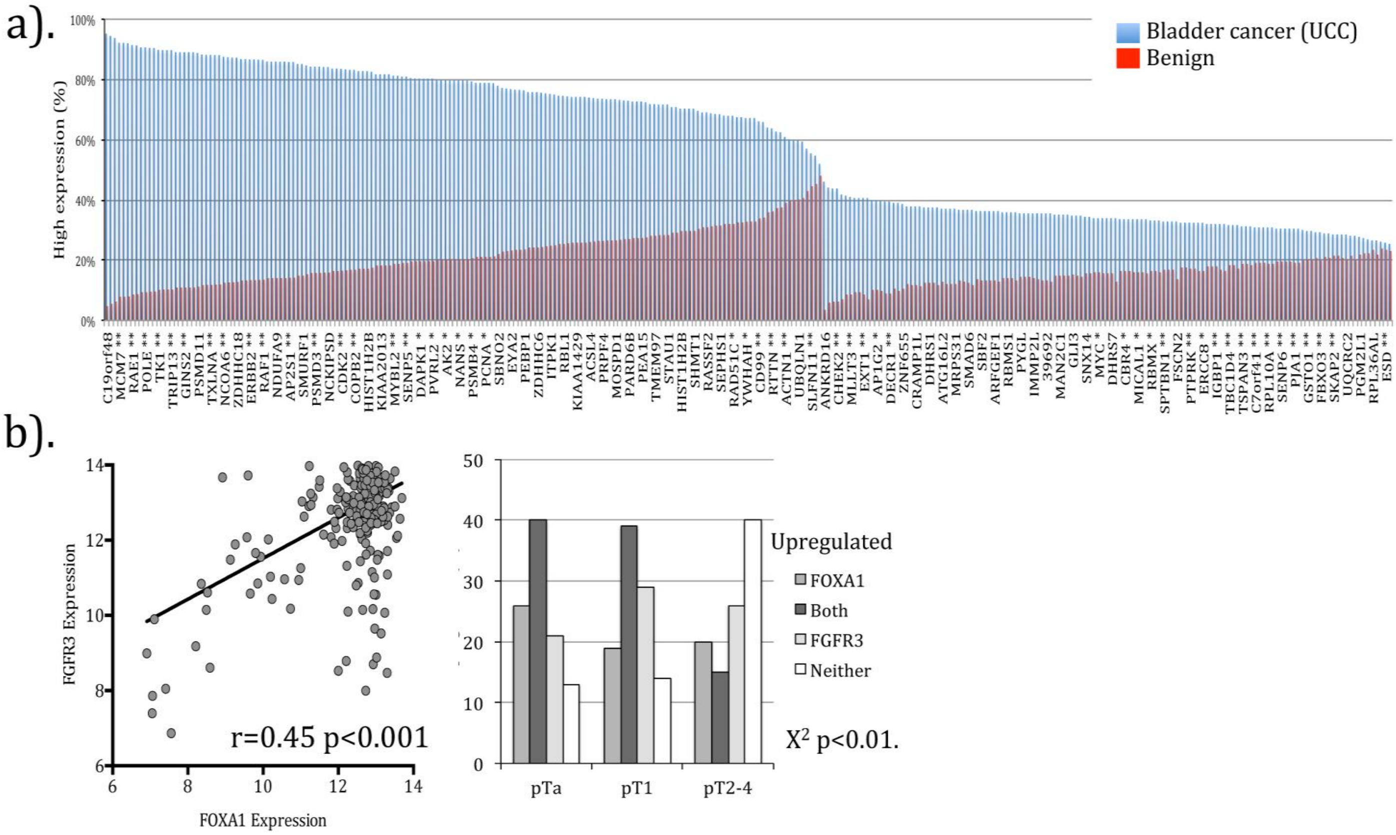

Supplementary figure 7: External validation of FOXA1, FGFR3 and correlated genes in UCC. We identified 295 genes in a reported microarray dataset [32] matching those with aberrant expression in tumors with FGFR3 mutation and cells with FOXA1 transfection. (a). The majority of these genes showed aberrant expression in malignant urothelial samples when compared to normal tissue (Chi sq. *p<0.5 and ** p<0.01). (b) Expression of FOXA1 and FGFR3 was correlated (r=0.45 (95%CI 0.35-0.55), p<0.001) and their upregulation occurred most frequently in non-muscle invasive (pTa (n=23) and pT1 (n=80)) tumors, when compared to invasive cancers (n=62). Chart show upregulation (around median value) for either, both or neither gene. (c). Significant differential expression was seen for 156/297 (53%) genes when pTa tumors were compared to pT1 (orange) and pT2-4 (blue). The color bar indicates the expected fold change as seen in FGFR3 mutant/FOXA1 transfected cells (green = down regulation, red = upregulation). (T Test *p<0.5 and ** p<0.01).
