# Supplementary material for: MicroRNA-99a and 100 mediated upregulation of FOXA1 in bladder cancer
Source: Oncotarget. 2014 Jul 15;5(15):6375–86. doi: 10.18632/oncotarget.2221 (PMC4171637; doi:10.18632/oncotarget.2221)
Supplement: Supplementary file 2 [file oncotarget-05-6375-s002.pdf]

Oncomine concept	Total genes	Top 10%	MiR-99a/100 potential targets	Microarray	Total Samples	Normal	Superficial/Non-muscle invasive	Infiltrating (pT2-4)	Squamous cell carcinoma	Unknown stage	
Superficial UCC	6,239	617	12	Custom	93	3	28	54	1	4	Blaveri et al. Clin Cancer Res 2005/06/01
Superficial vs. Normal urothelium	6,239	540	7	Custom	93						Blaveri et al. Clin Cancer Res 2005/06/01
Superficial UCC	4,564	429	9	HumanGeneFL Array (4564)	40		30	10			Dyrskjot et al. Nat Genet 2003/01/01
Superficial UCC	12,427	1,242	22	Human Genome U133A Array	60	14	33	13			Dyrskjot et al. Cancer Res 2004/06/01
Superficial vs. Normal urothelium	12,427	1,242	11	Human Genome U133A Array	60	14	33	13			Dyrskjot et al. Cancer Res 2004/06/01
Superficial UCC	589	58	3	GPL4060 (589)	808		706	100	2		Dyrskjot et al. Clin Cancer Res 2007/06/15
Superficial UCC	1,073	107	2	Custom	54	4	22	28			Modlich et al. Clin Cancer Res 2004/05/15
Superficial vs. Normal urothelium	1,073	107	2	Custom	54	4	22	28			Modlich et al. Clin Cancer Res 2004/05/15
Superficial UCC	12,427	1,242	10	Human Genome U133A Array	9		3	6			Modlich et al. Clin Cancer Res 2004/05/15
Superficial UCC	12,427	1,242	12	Human Genome U133A Array	157	48	28	81			Sanchez-Carbayo et al. J Clin Oncol 2006/02/10
Superficial vs. Normal urothelium	12,427	1,242	13	Human Genome U133A Array	157	48	28	81			Sanchez-Carbayo et al. J Clin Oncol 2006/02/10
Superficial UCC	7,820	725	14	Human Genome U95A-Av2 Array	57		25	32			Stransky et al. Nat Genet 2006/12/01

Supplementary table 1.

a). Reciprocal loss of microRNA expression following miRs-99a/100 knock-down

	miR-485-5p	miR-500a	miR-486	let-7e	miR-657	miR-133b	miR-139-5p
Fold change (with anti miR-100)	0.001	0.01	0.01	0.04	0.04	0.001	0.05
Fold change (with anti miR-99a)	0.001	0.01	0.06	0.27	0.33	0.99	0.05
Correlation with miR-100 (Pearson, R)	0.71	0.52	0.43	0.56	0.26	0.65	0.68
p value	2.01E-09	6.20E-06	4.98E-05	5.14E-08	9.52E-02	4.03E-11	2.38E-11
Correlation with miR-99a (Pearson, R)	0.68	0.57	0.46	0.6	0.27	0.65	0.74
p value	1.61E-08	7.60E-07	1.23E-05	4.61E-09	8.87E-02	4.12E-11	8.00E-14

b). Transcription factors within the FOXA1 promoter

	miR-485-5p	miR-500a	miR-486	let-7e	miR-657	miR-133b	miR-139-5p
AHR		1					
AML1A		1					
BACH1				1			
FOXL1	1						
FOXO1a			1				1
HOXA9				1		1	1
JunD	1						
SOX5	1						1
Sp1					1	1	

Supplementary table 2.

a), Genes with FDR <5% in FOXA1 transfected cells compared to controls

Probe Set ID	Gene Symbol	SAM Score(d)	Fold Change	FDR (q-value(%))	CpG Hypermethyaltion *	CpG Hypomethylation *
217010_s_at	CDC25C	3.49	1.77	4.39	0	5
1555526_a_at	38961	4.24	1.59	3.47	0	1
216248_s_at	NR4A2	3.4	1.57	4.39	0	2
205510_s_at	FLJ10038	4.34	1.52	3.47	1	2
203837_at	MAP3K5	3.72	1.5	3.47	1	0
244110_at	MLL	3.38	1.49	4.39	0	3
226875_at	DOCK11	3.23	1.49	4.39	0	0
203919_at	TCEA2	3.16	1.48	4.39	0	1
206204_at	GRB14	3.64	1.47	4.39	1	3
235435_at	AASDH	3.18	1.47	4.39	0	3
201631_s_at	IER3	3.31	1.47	4.39	1	0
217983_s_at	RNASET2	3.63	1.46	4.39	3	0
201452_at	RHEB	3.98	1.46	3.47	1	0
209754_s_at	TMPO	3.44	1.42	4.39	0	2
203367_at	DUSP14	-2.94	0.79	4.39	0	2
202251_at	PRPF3	-3.34	0.76	3.47	0	1
214315_x_at	CALR	-2.82	0.76	4.39	2	1
208130_s_at	TBXAS1	-2.83	0.76	4.39	1	2
1564674_a_at	CDC20B	-2.88	0.75	4.39	0	1
229044_at	NUDT17	-2.81	0.74	4.39	0	0
202644_s_at	TNFAIP3	-2.89	0.74	4.39	0	1
223691_at	RGS22	-3.02	0.74	4.39	0	1
209646_x_at	ALDH1B1	-2.84	0.74	4.39	0	3
202024_at	ASNA1	-2.98	0.73	4.39	1	1
230234_at	FXN	-2.93	0.73	4.39	0	2
232270_at	C9orf3	-2.92	0.73	4.39	0	1
209044_x_at	SF3B4	-2.88	0.72	4.39	0	2
219741_x_at	ZNF552	-3.1	0.71	4.39	0	4
217473_x_at	SLC11A1	-3.18	0.71	3.47	1	2
219993_at	SOX17	-2.91	0.7	4.39	1	1
235231_at	ZNF789	-3.15	0.69	3.47	1	2
216781_at	KIAA1751	-4.4	0.67	0	1	0
242727_at	ARL5B	-3.15	0.67	3.47	2	1
211697_x_at	PNO1	-3.38	0.67	3.47	0	5
203332_s_at	INPP5D	-3.11	0.67	3.47	0	0
222187_x_at	G3BP1	-3.31	0.67	3.47	0	2
229080_at	EMID2	-2.96	0.67	4.39	2	1
209852_x_at	PSME3	-2.9	0.66	4.39	2	1
210095_s_at	IGFBP3	-3.57	0.66	0	1	2
239041_at	HIST1H2AK	-3.89	0.62	0	1	2
219199_at	AFF4	-4.2	0.61	0	0	0
214041_x_at	RPL37A	-4.65	0.59	0	0	3
204602_at	DKK1	-4.86	0.58	0	2	0
214472_at	HIST1H2AD	-5.22	0.58	0	1	3
209806_at	HIST1H2BK	-3.68	0.58	0	1	4
205249_at	EGR2	-3.43	0.57	0	1	1
203914_x_at	HPGD	-3.11	0.57	3.47	0	1
214391_x_at	PTGER1	-4.97	0.47	0	0	2
229485_x_at	SHISA3	-5.45	0.22	0	0	2
211371_at	MAP2K5	-6.18	0.04	0	0	1

* CpG Scores reflects the sum of neighbouring 9 probes with either >90% (defined as hyper) or <10% (defined as hypo) enrichment for methyl cytosine

Supplementary table 3a.

b). Pathway enrichment analysis for genes with FDR <5% in FOXA1 transfected cells compared to controls (selected for statistical significance)

Annotation Cluster 1	Enrichment Score: 1.8978752402573393										
Category	Term	Count	P Value	Genes	List Total	Pop Hits	Pop Total	Fold Enrichment	Bonferroni	Benjamini	FDR
GOTERM_BP_FAT	GO:0006690~icosanoid metabolic process	3	0.01	TBXAS1, HPGD, C9ORF3	42	47	13528	20.56	1.00	0.88	12.63
GOTERM_BP_FAT	GO:0033559~unsaturated fatty acid metabolic process	3	0.01	TBXAS1, HPGD, C9ORF3	42	51	13528	18.95	1.00	0.71	14.63
GOTERM_BP_FAT	GO:0006631~fatty acid metabolic process	4	0.02	TBXAS1, HPGD, C9ORF3, AASDH	42	198	13528	6.51	1.00	0.83	28.46
Annotation Cluster 2	Enrichment Score: 1.5030983730982628										
Category	Term	Count	PValue	Genes	List Total	Pop Hits	Pop Total	Fold Enrichment	Bonferroni	Benjamini	FDR
UP_SEQ_FEATURE	cross-link:Glycyl lysine isopeptide (Lys-Gly) (interchain with G-Cter in ubiquitin)	5	0.00	MLL, HIST1H2BK, HIST1H2AD, HIST1H2AK, TCEA2	48	199	19113	10.00	0.29	0.16	1.83
SP_PIR_KEYWORDS	methylation	5	0.00	HIST1H2BK, HIST1H2AD, G3BP1, RHEB, HIST1H2AK	48	242	19235	8.28	0.35	0.19	3.35
SP_PIR_KEYWORDS	chromosomal protein	4	0.01	HIST1H2BK, HIST1H2AD, HIST1H2AK, TMPO	48	145	19235	11.05	0.55	0.15	6.19
SP_PIR_KEYWORDS	nucleosome core	3	0.01	HIST1H2BK, HIST1H2AD, HIST1H2AK	48	48	19235	25.05	0.60	0.14	7.08
INTERPRO	IPR007125:Histone core	3	0.01	HIST1H2BK, HIST1H2AD, HIST1H2AK	45	45	16659	24.68	0.60	0.60	7.21
SP_PIR_KEYWORDS	isopeptide bond	5	0.01	MLL, HIST1H2BK, HIST1H2AD, HIST1H2AK, TCEA2	48	319	19235	6.28	0.68	0.13	8.65
INTERPRO	IPR009072:Histone-fold	3	0.01	HIST1H2BK, HIST1H2AD, HIST1H2AK	45	54	16659	20.57	0.73	0.48	10.13
GOTERM_CC_FAT	GO:0000786~nucleosome	3	0.01	HIST1H2BK, HIST1H2AD, HIST1H2AK	32	63	12782	19.02	0.60	0.60	10.52
GOTERM_CC_FAT	GO:0000785~chromatin	4	0.01	HIST1H2BK, HIST1H2AD, HIST1H2AK, TMPO	32	200	12782	7.99	0.68	0.43	12.61
GOTERM_CC_FAT	GO:0032993~protein-DNA complex	3	0.02	HIST1H2BK, HIST1H2AD, HIST1H2AK	32	86	12782	13.93	0.81	0.43	18.27
GOTERM_BP_FAT	GO:0006334~nucleosome assembly	3	0.03	HIST1H2BK, HIST1H2AD, HIST1H2AK	42	84	13528	11.50	1.00	0.83	33.60
GOTERM_BP_FAT	GO:0031497~chromatin assembly	3	0.03	HIST1H2BK, HIST1H2AD, HIST1H2AK	42	87	13528	11.11	1.00	0.80	35.43
GOTERM_BP_FAT	GO:0065004~protein-DNA complex assembly	3	0.03	HIST1H2BK, HIST1H2AD, HIST1H2AK	42	91	13528	10.62	1.00	0.80	37.86
GOTERM_BP_FAT	GO:0034728~nucleosome organization	3	0.03	HIST1H2BK, HIST1H2AD, HIST1H2AK	42	93	13528	10.39	1.00	0.79	39.07
GOTERM_BP_FAT	GO:0006323~DNA packaging	3	0.05	HIST1H2BK, HIST1H2AD, HIST1H2AK	42	117	13528	8.26	1.00	0.83	53.12
Annotation Cluster 3	Enrichment Score: 1.253161046578192										
Category	Term	Count	PValue	Genes	List Total	Pop Hits	Pop Total	Fold Enrichment	Bonferroni	Benjamini	FDR
GOTERM_BP_FAT	GO:0042981~regulation of apoptosis	8	0.01	IER3, MAP3K5, NR4A2, PSME3, INPP5D, TNFAIP3, CALR, IGFBP3	42	804	13528	3.20	1.00	0.83	13.78
GOTERM_BP_FAT	GO:0043067~regulation of programmed cell death	8	0.01	IER3, MAP3K5, NR4A2, PSME3, INPP5D, TNFAIP3, CALR, IGFBP3	42	812	13528	3.17	1.00	0.77	14.46
GOTERM_BP_FAT	GO:0010941~regulation of cell death	8	0.01	IER3, MAP3K5, NR4A2, PSME3, INPP5D, TNFAIP3, CALR, IGFBP3	42	815	13528	3.16	1.00	0.66	14.72
SP_PIR_KEYWORDS	Apoptosis	5	0.01	MAP3K5, MLL, PSME3, INPP5D, TNFAIP3	48	381	19235	5.26	0.87	0.19	15.30
GOTERM_BP_FAT	GO:0016265~death	7	0.02	IER3, MAP3K5, MLL, NR4A2, PSME3, INPP5D, TNFAIP3	42	724	13528	3.11	1.00	0.85	27.34
GOTERM_BP_FAT	GO:0006915~apoptosis	6	0.03	IER3, MAP3K5, MLL, PSME3, INPP5D, TNFAIP3	42	602	13528	3.21	1.00	0.79	41.02
GOTERM_BP_FAT	GO:0012501~programmed cell death	6	0.04	IER3, MAP3K5, MLL, PSME3, INPP5D, TNFAIP3	42	611	13528	3.16	1.00	0.77	42.77
Annotation Cluster 4	Enrichment Score: 1.1924683089446422										
Category	Term	Count	PValue	Genes	List Total	Pop Hits	Pop Total	Fold Enrichment	Bonferroni	Benjamini	FDR
GOTERM_BP_FAT	GO:0042981~regulation of apoptosis	8	0.01	IER3, MAP3K5, NR4A2, PSME3, INPP5D, TNFAIP3, CALR, IGFBP3	42	804	13528	3.20	1.00	0.83	13.78
GOTERM_BP_FAT	GO:0043067~regulation of programmed cell death	8	0.01	IER3, MAP3K5, NR4A2, PSME3, INPP5D, TNFAIP3, CALR, IGFBP3	42	812	13528	3.17	1.00	0.77	14.46
GOTERM_BP_FAT	GO:0010941~regulation of cell death	8	0.01	IER3, MAP3K5, NR4A2, PSME3, INPP5D, TNFAIP3, CALR, IGFBP3	42	815	13528	3.16	1.00	0.66	14.72
Annotation Cluster 5	Enrichment Score: 1.0901952415239662										
Category	Term	Count	PValue	Genes	List Total	Pop Hits	Pop Total	Fold Enrichment	Bonferroni	Benjamini	FDR
GOTERM_CC_FAT	GO:0070013~intracellular organelle lumen	10	0.02	MLL, FXN, ALDH1B1, PNO1, PRPF3, TCEA2, CDC25C, ASNA1, CALR, HPGD	32	1779	12782	2.25	0.87	0.39	21.39
GOTERM_CC_FAT	GO:0043233~organelle lumen	10	0.03	MLL, FXN, ALDH1B1, PNO1, PRPF3, TCEA2, CDC25C, ASNA1, CALR, HPGD	32	1820	12782	2.19	0.90	0.37	24.13
GOTERM_CC_FAT	GO:0031974~membrane-enclosed lumen	10	0.03	MLL, FXN, ALDH1B1, PNO1, PRPF3, TCEA2, CDC25C, ASNA1, CALR, HPGD	32	1856	12782	2.15	0.93	0.35	26.69
Annotation Cluster 6	Enrichment Score: 1.0677213174351998										
Category	Term	Count	PValue	Genes	List Total	Pop Hits	Pop Total	Fold Enrichment	Bonferroni	Benjamini	FDR
GOTERM_BP_FAT	GO:0006873~cellular ion homeostasis	5	0.03	SLC11A1, EGR2, FXN, ASNA1, CALR	42	374	13528	4.31	1.00	0.85	32.90
GOTERM_BP_FAT	GO:0055082~cellular chemical homeostasis	5	0.03	SLC11A1, EGR2, FXN, ASNA1, CALR	42	380	13528	4.24	1.00	0.81	34.31
GOTERM_BP_FAT	GO:0050801~ion homeostasis	5	0.03	SLC11A1, EGR2, FXN, ASNA1, CALR	42	409	13528	3.94	1.00	0.77	41.28
GOTERM_BP_FAT	GO:0030003~cellular cation homeostasis	4	0.04	SLC11A1, FXN, ASNA1, CALR	42	254	13528	5.07	1.00	0.80	47.17
Annotation Cluster 7	Enrichment Score: 0.8889030478932497										
Category	Term	Count	PValue	Genes	List Total	Pop Hits	Pop Total	Fold Enrichment	Bonferroni	Benjamini	FDR
SP_PIR_KEYWORDS	dna-binding	13	0.00	EGR2, ZNF552, MLL, HIST1H2AD, G3BP1, NR4A2, ZNF789, HIST1H2BK, HIST1H2AK, TMPO, SOX17, TCEA2, TNFAIP3	48	1868	19235	2.79	0.19	0.19	1.67
SP_PIR_KEYWORDS	metal-binding	16	0.00	NUDT17, EGR2, ZNF552, TBXAS1, MLL, NR4A2, CALR, C9ORF3, MAP3K5, ZNF789, RHEB, RPL37A, TCEA2, TNFAIP3, ASNA1, MAP2K5	48	2972	19235	2.16	0.42	0.17	4.24
SP_PIR_KEYWORDS	nucleus	20	0.00	EGR2, ZNF552, MLL, HIST1H2AD, PNO1, G3BP1, NR4A2, AFF4, PRPF3, CDC25C, SF3B4, ZNF789, HIST1H2BK, HIST1H2AK, PSME3, TMPO, SOX17, TCEA2, TNFAIP3, ASNA1	48	4283	19235	1.87	0.44	0.14	4.55
SP_PIR_KEYWORDS	zinc finger	4	0.01	MLL, RPL37A, TCEA2, TNFAIP3	48	153	19235	10.48	0.60	0.12	7.14
GOTERM_MF_FAT	GO:0003677~DNA binding	15	0.01	EGR2, ZNF552, MLL, HIST1H2AD, G3BP1, AFF4, NR4A2, CALR, ZNF789, HIST1H2BK, HIST1H2AK, TMPO, SOX17, TCEA2, TNFAIP3	42	2331	12983	1.99	0.84	0.84	11.31
SP_PIR_KEYWORDS	zinc	11	0.04	MLL, ZNF789, ZNF552, EGR2, NR4A2, RPL37A, TCEA2, TNFAIP3, ASNA1, CALR, C9ORF3	48	2189	19235	2.01	1.00	0.37	35.51

Supplementary table 3b.

Antibodies used in Western Blots	Manufacturer		Dilution
FGFR3	Cell Signalling	#3160	1000
p-ERK	Cell Signalling	#4670	1000
!GF1R	Cell Signalling	#3027	1000
FOXA1	Abcam	ab55178	1000
HOXA1	R&D Systems	AF5014	100
Beta Actin	Sigma	A5361	2000

Supplemntary table 4

a), Genes with FDR <5% in FOXA1 transfected cells compared to controls

Probe Set ID	Gene Symbol	SAM Score(d)	Fold Change	FDR (q-value(%))	CpG Hypermethyaltion *	CpG Hypomethylation *
217010_s_at	CDC25C	3.49	1.77	4.39	0	5
1555526_a_at	38961	4.24	1.59	3.47	0	1
216248_s_at	NR4A2	3.4	1.57	4.39	0	2
205510_s_at	FLJ10038	4.34	1.52	3.47	1	2
203837_at	MAP3K5	3.72	1.5	3.47	1	0
244110_at	MLL	3.38	1.49	4.39	0	3
226875_at	DOCK11	3.23	1.49	4.39	0	0
203919_at	TCEA2	3.16	1.48	4.39	0	1
206204_at	GRB14	3.64	1.47	4.39	1	3
235435_at	AASDH	3.18	1.47	4.39	0	3
201631_s_at	IER3	3.31	1.47	4.39	1	0
217983_s_at	RNASET2	3.63	1.46	4.39	3	0
201452_at	RHEB	3.98	1.46	3.47	1	0
209754_s_at	TMPO	3.44	1.42	4.39	0	2
203367_at	DUSP14	-2.94	0.79	4.39	0	2
202251_at	PRPF3	-3.34	0.76	3.47	0	1
214315_x_at	CALR	-2.82	0.76	4.39	2	1
208130_s_at	TBXAS1	-2.83	0.76	4.39	1	2
1564674_a_at	CDC20B	-2.88	0.75	4.39	0	1
229044_at	NUDT17	-2.81	0.74	4.39	0	0
202644_s_at	TNFAIP3	-2.89	0.74	4.39	0	1
223691_at	RGS22	-3.02	0.74	4.39	0	1
209646_x_at	ALDH1B1	-2.84	0.74	4.39	0	3
202024_at	ASNA1	-2.98	0.73	4.39	1	1
230234_at	FXN	-2.93	0.73	4.39	0	2
232270_at	C9orf3	-2.92	0.73	4.39	0	1
209044_x_at	SF3B4	-2.88	0.72	4.39	0	2
219741_x_at	ZNF552	-3.1	0.71	4.39	0	4
217473_x_at	SLC11A1	-3.18	0.71	3.47	1	2
219993_at	SOX17	-2.91	0.7	4.39	1	1
235231_at	ZNF789	-3.15	0.69	3.47	1	2
216781_at	KIAA1751	-4.4	0.67	0	1	0
242727_at	ARL5B	-3.15	0.67	3.47	2	1
211697_x_at	PNO1	-3.38	0.67	3.47	0	5
203332_s_at	INPP5D	-3.11	0.67	3.47	0	0
222187_x_at	G3BP1	-3.31	0.67	3.47	0	2
229080_at	EMID2	-2.96	0.67	4.39	2	1
209852_x_at	PSME3	-2.9	0.66	4.39	2	1
210095_s_at	IGFBP3	-3.57	0.66	0	1	2
239041_at	HIST1H2AK	-3.89	0.62	0	1	2
219199_at	AFF4	-4.2	0.61	0	0	0
214041_x_at	RPL37A	-4.65	0.59	0	0	3
204602_at	DKK1	-4.86	0.58	0	2	0
214472_at	HIST1H2AD	-5.22	0.58	0	1	3
209806_at	HIST1H2BK	-3.68	0.58	0	1	4
205249_at	EGR2	-3.43	0.57	0	1	1
203914_x_at	HPGD	-3.11	0.57	3.47	0	1
214391_x_at	PTGER1	-4.97	0.47	0	0	2
229485_x_at	SHISA3	-5.45	0.22	0	0	2
211371_at	MAP2K5	-6.18	0.04	0	0	1

* CpG Scores reflects the sum of neighbouring 9 probes with either >90% (defined as hyper) or <10% (defined as hypo) enrichment for methyl cytosine

Supplementary table 2a.

b). Pathway enrichment analysis for genes with FDR <5% in FOXA1 transfected cells compared to controls (selected for statistical significance)

Annotation Cluster 1	Enrichment Score: 1.8978752402573393										
Category	Term	Count	P Value	Genes	List Total	Pop Hits	Pop Total	Fold Enrichment	Bonferroni	Benjamini	FDR
GOTERM_BP_FAT	GO:0006690~icosanoid metabolic process	3	0.01	TBXAS1, HPGD, C9ORF3	42	47	13528	20.56	1.00	0.88	12.63
GOTERM_BP_FAT	GO:0033559~unsaturated fatty acid metabolic process	3	0.01	TBXAS1, HPGD, C9ORF3	42	51	13528	18.95	1.00	0.71	14.63
GOTERM_BP_FAT	GO:0006631~fatty acid metabolic process	4	0.02	TBXAS1, HPGD, C9ORF3, AASDH	42	198	13528	6.51	1.00	0.83	28.46
Annotation Cluster 2	Enrichment Score: 1.5030983730982628										
Category	Term	Count	PValue	Genes	List Total	Pop Hits	Pop Total	Fold Enrichment	Bonferroni	Benjamini	FDR
UP_SEQ_FEATURE	cross-link:Glycyl lysine isopeptide (Lys-Gly) (interchain with G-Cter in ubiquitin)	5	0.00	MLL, HIST1H2BK, HIST1H2AD, HIST1H2AK, TCEA2	48	199	19113	10.00	0.29	0.16	1.83
SP_PIR_KEYWORDS	methylation	5	0.00	HIST1H2BK, HIST1H2AD, G3BP1, RHEB, HIST1H2AK	48	242	19235	8.28	0.35	0.19	3.35
SP_PIR_KEYWORDS	chromosomal protein	4	0.01	HIST1H2BK, HIST1H2AD, HIST1H2AK, TMPO	48	145	19235	11.05	0.55	0.15	6.19
SP_PIR_KEYWORDS	nucleosome core	3	0.01	HIST1H2BK, HIST1H2AD, HIST1H2AK	48	48	19235	25.05	0.60	0.14	7.08
INTERPRO	IPR007125:Histone core	3	0.01	HIST1H2BK, HIST1H2AD, HIST1H2AK	45	45	16659	24.68	0.60	0.60	7.21
SP_PIR_KEYWORDS	isopeptide bond	5	0.01	MLL, HIST1H2BK, HIST1H2AD, HIST1H2AK, TCEA2	48	319	19235	6.28	0.68	0.13	8.65
INTERPRO	IPR009072:Histone-fold	3	0.01	HIST1H2BK, HIST1H2AD, HIST1H2AK	45	54	16659	20.57	0.73	0.48	10.13
GOTERM_CC_FAT	GO:0000786~nucleosome	3	0.01	HIST1H2BK, HIST1H2AD, HIST1H2AK	32	63	12782	19.02	0.60	0.60	10.52
GOTERM_CC_FAT	GO:0000785~chromatin	4	0.01	HIST1H2BK, HIST1H2AD, HIST1H2AK, TMPO	32	200	12782	7.99	0.68	0.43	12.61
GOTERM_CC_FAT	GO:0032993~protein-DNA complex	3	0.02	HIST1H2BK, HIST1H2AD, HIST1H2AK	32	86	12782	13.93	0.81	0.43	18.27
GOTERM_BP_FAT	GO:0006334~nucleosome assembly	3	0.03	HIST1H2BK, HIST1H2AD, HIST1H2AK	42	84	13528	11.50	1.00	0.83	33.60
GOTERM_BP_FAT	GO:0031497~chromatin assembly	3	0.03	HIST1H2BK, HIST1H2AD, HIST1H2AK	42	87	13528	11.11	1.00	0.80	35.43
GOTERM_BP_FAT	GO:0065004~protein-DNA complex assembly	3	0.03	HIST1H2BK, HIST1H2AD, HIST1H2AK	42	91	13528	10.62	1.00	0.80	37.86
GOTERM_BP_FAT	GO:0034728~nucleosome organization	3	0.03	HIST1H2BK, HIST1H2AD, HIST1H2AK	42	93	13528	10.39	1.00	0.79	39.07
GOTERM_BP_FAT	GO:0006323~DNA packaging	3	0.05	HIST1H2BK, HIST1H2AD, HIST1H2AK	42	117	13528	8.26	1.00	0.83	53.12
Annotation Cluster 3	Enrichment Score: 1.253161046578192										
Category	Term	Count	PValue	Genes	List Total	Pop Hits	Pop Total	Fold Enrichment	Bonferroni	Benjamini	FDR
GOTERM_BP_FAT	GO:0042981~regulation of apoptosis	8	0.01	IER3, MAP3K5, NR4A2, PSME3, INPP5D, TNFAIP3, CALR, IGFBP3	42	804	13528	3.20	1.00	0.83	13.78
GOTERM_BP_FAT	GO:0043067~regulation of programmed cell death	8	0.01	IER3, MAP3K5, NR4A2, PSME3, INPP5D, TNFAIP3, CALR, IGFBP3	42	812	13528	3.17	1.00	0.77	14.46
GOTERM_BP_FAT	GO:0010941~regulation of cell death	8	0.01	IER3, MAP3K5, NR4A2, PSME3, INPP5D, TNFAIP3, CALR, IGFBP3	42	815	13528	3.16	1.00	0.66	14.72
SP_PIR_KEYWORDS	Apoptosis	5	0.01	MAP3K5, MLL, PSME3, INPP5D, TNFAIP3	48	381	19235	5.26	0.87	0.19	15.30
GOTERM_BP_FAT	GO:0016265~death	7	0.02	IER3, MAP3K5, MLL, NR4A2, PSME3, INPP5D, TNFAIP3	42	724	13528	3.11	1.00	0.85	27.34
GOTERM_BP_FAT	GO:0006915~apoptosis	6	0.03	IER3, MAP3K5, MLL, PSME3, INPP5D, TNFAIP3	42	602	13528	3.21	1.00	0.79	41.02
GOTERM_BP_FAT	GO:0012501~programmed cell death	6	0.04	IER3, MAP3K5, MLL, PSME3, INPP5D, TNFAIP3	42	611	13528	3.16	1.00	0.77	42.77
Annotation Cluster 4	Enrichment Score: 1.1924683089446422										
Category	Term	Count	PValue	Genes	List Total	Pop Hits	Pop Total	Fold Enrichment	Bonferroni	Benjamini	FDR
GOTERM_BP_FAT	GO:0042981~regulation of apoptosis	8	0.01	IER3, MAP3K5, NR4A2, PSME3, INPP5D, TNFAIP3, CALR, IGFBP3	42	804	13528	3.20	1.00	0.83	13.78
GOTERM_BP_FAT	GO:0043067~regulation of programmed cell death	8	0.01	IER3, MAP3K5, NR4A2, PSME3, INPP5D, TNFAIP3, CALR, IGFBP3	42	812	13528	3.17	1.00	0.77	14.46
GOTERM_BP_FAT	GO:0010941~regulation of cell death	8	0.01	IER3, MAP3K5, NR4A2, PSME3, INPP5D, TNFAIP3, CALR, IGFBP3	42	815	13528	3.16	1.00	0.66	14.72
Annotation Cluster 5	Enrichment Score: 1.0901952415239662										
Category	Term	Count	PValue	Genes	List Total	Pop Hits	Pop Total	Fold Enrichment	Bonferroni	Benjamini	FDR
GOTERM_CC_FAT	GO:0070013~intracellular organelle lumen	10	0.02	MLL, FXN, ALDH1B1, PNO1, PRPF3, TCEA2, CDC25C, ASNA1, CALR, HPGD	32	1779	12782	2.25	0.87	0.39	21.39
GOTERM_CC_FAT	GO:0043233~organelle lumen	10	0.03	MLL, FXN, ALDH1B1, PNO1, PRPF3, TCEA2, CDC25C, ASNA1, CALR, HPGD	32	1820	12782	2.19	0.90	0.37	24.13
GOTERM_CC_FAT	GO:0031974~membrane-enclosed lumen	10	0.03	MLL, FXN, ALDH1B1, PNO1, PRPF3, TCEA2, CDC25C, ASNA1, CALR, HPGD	32	1856	12782	2.15	0.93	0.35	26.69
Annotation Cluster 6	Enrichment Score: 1.0677213174351998										
Category	Term	Count	PValue	Genes	List Total	Pop Hits	Pop Total	Fold Enrichment	Bonferroni	Benjamini	FDR
GOTERM_BP_FAT	GO:0006873~cellular ion homeostasis	5	0.03	SLC11A1, EGR2, FXN, ASNA1, CALR	42	374	13528	4.31	1.00	0.85	32.90
GOTERM_BP_FAT	GO:0055082~cellular chemical homeostasis	5	0.03	SLC11A1, EGR2, FXN, ASNA1, CALR	42	380	13528	4.24	1.00	0.81	34.31
GOTERM_BP_FAT	GO:0050801~ion homeostasis	5	0.03	SLC11A1, EGR2, FXN, ASNA1, CALR	42	409	13528	3.94	1.00	0.77	41.28
GOTERM_BP_FAT	GO:0030003~cellular cation homeostasis	4	0.04	SLC11A1, FXN, ASNA1, CALR	42	254	13528	5.07	1.00	0.80	47.17
Annotation Cluster 7	Enrichment Score: 0.8889030478932497										
Category	Term	Count	PValue	Genes	List Total	Pop Hits	Pop Total	Fold Enrichment	Bonferroni	Benjamini	FDR
SP_PIR_KEYWORDS	dna-binding	13	0.00	EGR2, ZNF552, MLL, HIST1H2AD, G3BP1, NR4A2, ZNF789, HIST1H2BK, HIST1H2AK, TMPO, SOX17, TCEA2, TNFAIP3	48	1868	19235	2.79	0.19	0.19	1.67
SP_PIR_KEYWORDS	metal-binding	16	0.00	NUDT17, EGR2, ZNF552, TBXAS1, MLL, NR4A2, CALR, C9ORF3, MAP3K5, ZNF789, RHEB, RPL37A, TCEA2, TNFAIP3, ASNA1, MAP2K5	48	2972	19235	2.16	0.42	0.17	4.24
SP_PIR_KEYWORDS	nucleus	20	0.00	EGR2, ZNF552, MLL, HIST1H2AD, PNO1, G3BP1, NR4A2, AFF4, PRPF3, CDC25C, SF3B4, ZNF789, HIST1H2BK, HIST1H2AK, PSME3, TMPO, SOX17, TCEA2, TNFAIP3, ASNA1	48	4283	19235	1.87	0.44	0.14	4.55
SP_PIR_KEYWORDS	zinc finger	4	0.01	MLL, RPL37A, TCEA2, TNFAIP3	48	153	19235	10.48	0.60	0.12	7.14
GOTERM_MF_FAT	GO:0003677~DNA binding	15	0.01	EGR2, ZNF552, MLL, HIST1H2AD, G3BP1, AFF4, NR4A2, CALR, ZNF789, HIST1H2BK, HIST1H2AK, TMPO, SOX17, TCEA2, TNFAIP3	42	2331	12983	1.99	0.84	0.84	11.31
SP_PIR_KEYWORDS	zinc	11	0.04	MLL, ZNF789, ZNF552, EGR2, NR4A2, RPL37A, TCEA2, TNFAIP3, ASNA1, CALR, C9ORF3	48	2189	19235	2.01	1.00	0.37	35.51

Supplementary table 2b.

Antibodies used in Western Blot:	Manufacturer		Dilution
FGFR3	Cell Signalling	#3160	1000
p-ERK	Cell Signalling	#4670	1000
!GF1R	Cell Signalling	#3027	1000
FOXA1	Abcam	ab55178	1000
HOXA1	R&D Systems	AF5014	100
Beta Actin	Sigma	A5361	2000

Supplementary table 3
